# The role of causal inference in health services research II: a framework for causal inference

**DOI:** 10.1007/s00038-020-01334-1

**Published:** 2020-02-12

**Authors:** André Moser, Milo A. Puhan, Marcel Zwahlen

**Affiliations:** 1 Epidemiology, Biostatistics and Prevention Institute, University of Zurich, Hirschengraben 84, 8001 Zurich, Switzerland; 2 Institute of Social and Preventive Medicine, University of Bern, Mittelstrasse 43, 3012 Bern, Switzerland

## Introduction

In a previous *Hints and Kinks*, we discussed the role of causal inference in tasks of health services research (HSR) using examples from health system interventions (Moser et al. 2020). In the present *Hints and Kinks*, we more formally introduce a principled framework for causal inference. Specifically, we discuss in more detail the role of counterfactuals for the definition of a causal effect and the ‘association is not causation’ adage. We continue on the example of a hospital merger (HM) as a health system intervention.

### Counterfactuals and causal effect

We introduced counterfactuals as hypothetical outcomes which are actually not observed in a real-world setting (Hernán 2004). We used an example of a HM, where we were interested in the causal question whether a HM reduces hospital readmissions (Moser et al. 2020). To answer this question, we need to define a causal effect, a statistical measure which relates probabilities of hospital readmissions when (1) every patient is treated under the situation of a HM versus (2) the HM *would not have been* implemented. Note that we never observe one of the two situations, because either the HM is implemented or not, but not both. We now introduce a formal notation for causal inference which allows us to mathematically define a causal effect.

For each patient, we would like to know his or her outcome (here, a hospital readmission) if the HM had not been implemented (denoted as *Y*^noHM^) together with the outcome under the HM (denoted as *Y*^HM^). The *superscripts* denote the counterfactual outcomes we can formalize, but which are actually not observed: Only *Y*^HM^ can be observed if the HM is implemented. An *average causal effect* in the study population can then be defined by the risk difference Probability(*Y*^HM^  = 1)–Probability(*Y*^noHM^  = 1), abbreviated as RD^Causal^. Note that we could also use other risk measures, for example a relative risk, for the definition of a causal effect. The choice of the used effect measure depends on the research question because the underlying scale (i.e., an additive scale for a risk difference or multiplicative scale for a risk ratio) influences its final interpretation (Hernán and Robins 2020).

An important question remains: How can we assess an effect measure based on outcomes which are actually not observed? One could compare the outcomes in the region with HM to outcomes in a 'control' region with no HM. Table 1 shows hypothetical patients with (known) counterfactual outcomes and actually observed outcomes (denoted with *subscriptsY*_noHM_, *Y*_HM_, *Y*_Observed_). For example, the patient with ID 5 was treated in the HM region with no observed hospital readmission (*Y*_Observed_ = 0). The observed outcome is equal to the counterfactual outcome in the HM region (*Y*_Observed_ = *Y*_HM_ = *Y*^HM^ = 0). Note that if this patient would have been treated in the control region, he or she would have had a readmission (*Y*^noHM^  = 1). Because this patient is actually only observed in the HM region, one will never observe the outcome of the control region (*Y*_noHM_ is missing). The mathematical notation for counterfactuals might be initially confusing, yet it is a necessary component for a causal inference framework.

**Table 1:** Study population of five patients

ID	Region	*Y*^noHM^	*Y*^HM^	*Y*_noHM_	*Y*_HM_	*Y*_Observed_
1	No HM	0	1	0	NA	0
2	No HM	0	1	0	NA	0
3	No HM	1	1	1	NA	1
4	HM	1	0	NA	0	0
**5**	**HM**	**1**	**0**	**NA**	**0**	**0**

*HM* Hospital merger, *NA* Not available, *Y*^noHM^ Counterfactual outcome in the region with no HM, *Y*^*HM*^ Counterfactual outcome in the HM region, *Y*_*noHM*_ Counterfactual outcome in the region with no HM, actually observed in the real world, *Y*_*HM*_ Counterfactual outcome in the HM region, actually observed in the real world, *Y*_*Observed*_ Observed outcome

In bold: the patient described in the manuscript

What is the average causal effect in the study population from Table 1? We get that the risk difference RD^Causal^ is zero, because Probability(*Y*^HM^ = 1) = 3/5 and Probability(*Y*^noHM^  = 1) = 3/5. Thus, the HM does not reduce hospital readmissions.

### Association versus causation

**Fig. 1:**
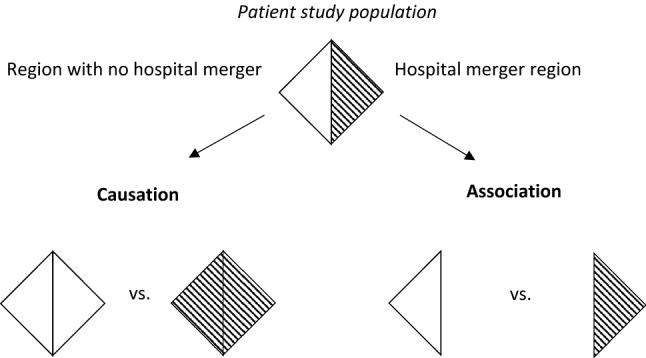
Graphical explanation of ‘association versus causation’ using the example of a hospital merger as a health system intervention. Study outcome: Hospital readmissions. ‘Association’ compares relationships in subsets of a study population, indicated by the separated triangles. For example, one compares the risk of hospital readmissions among patients treated in a region with a hospital merger and among patients treated in a region without a hospital merger. ‘Causation’ compares situations (i.e. ‘what-if’ questions) between hypothetical study populations. For example, one compares hospital readmissions in a population where every patient would have been treated in a region with a hospital merger with a population where every patient would have been treated in the same region, but without a hospital merger *Source*: Figure adapted from Hernán (2004)

An *associational effect measure* generally compares risks in subsets of a study population by conditioning on certain study characteristics (see Fig. 1) (Hernán 2004). In the example of Table 1, one relates the risk of hospital readmissions *among patients in the HM region* with the risk *among patients in the control region*. Let us define

$$\begin{gathered} {\text{RD}}^{{{\text{Associational}}}} := {\text{Probability}}\left( {Y^{{{\text{Observed}}}} = {1\text{ among patients in the HM region}}} \right) \hfill \\ - {\text{Probability}}\left( {Y^{{{\text{Observed}}}} = {1\text{ among patients in the control region}}} \right), \hfill \\ \end{gathered}$$

as the associational risk difference in the study population. We obtain from Table 1 that the first expression of RD^Associational^ is 0 (two patients were treated in the HM region without an observed hospital readmission) and the second expression 1/3 (three patients were treated in the control region with one hospital readmission). Thus, RD^Associational^ is equal to 0–1/3 = –1/3, i.e., the risk of hospital readmissions in the HM region is lower compared to the risk in the control region.

The difference between the derived causal effect RD^Causal^ and the associational effect RD^Associational^ leads to the famous ‘association is not causation’ adage. Likely because of this adage, many researchers in HSR avoid any causal terminology, especially when they use ‘only’ observational data (Hernán 2018). They argue that the above comparison of outcomes between an ‘intervention’ and a ‘control’ region does not allow for any causal conclusions because the regions differ in several ways, for example, due to the case mix of treated patients, the skill-grade mix of medical personnel or the availability of health care services. When a study design randomly allocates patients before hospital entry to either the HM region or the control region (and patients and health care providers perfectly comply with that assignment), researchers would interpret statistical findings as causal. But in fact, many studies in HSR are observational studies without a random allocation of patients to treatment groups. Still, often only ‘descriptive’ and ‘modeling’ approaches are then used to support decision-making in health systems, even if the background is inherently causal. Whether the reported effect measure should be used from a causal inference approach or from descriptive and modeling approaches strongly depends on the intended HSR question.

How can researchers integrate ‘causality’ in HSR? Our above introduced components of a framework for causal inference is the backbone for modern causal inference. Modern causal inference allows for inference which mimics a situation as if patients would have been assigned by random allocation, despite using an observational study design. Topics for recent calls of causal inference approaches in HSR include, for example, comparative effectiveness research, payment scheme evaluations, health care utilization or the use of simulation studies (see Table 2). Principles of modern causal inference are described and explained in several textbooks (van der Laan and Sherri 2011; Pearl et al. 2016; Hernán and Robins 2020).

**Table 2:** Selected study examples using causal inferences approaches in health services research

Name	Topic	Type of data	Country	Study years
Danaei et al. (2018)	Treatment strategies of statins	Electronic health records	United Kingdom	2000–2010
Dickerman et al. (2019)	Benefit–harm assessment of statins in cancer patients	Electronic health records	United Kingdom	1999–2015
García-Albéniz et al. (2017)	Colorectal cancer screening	Insurance claims data	United States of America	2005–2012
Gaughan et al. (2019)	Payment scheme evaluation of same-day discharges	Administrative data	United Kingdom	2006–2014
Héroux et al. (2014)	Primary care utilization	Health insurance data	Canada	2002–2005
Kuehne et al. (2019)	Comparative effectiveness of statins	Health insurance data	Not specified	Not specified
Murray et al. (2018)	Calibration targets of simulation models	Simulation models	Multinational	2010–2013
Neugebauer et al. (2012)	Comparative effectiveness of diabetes treatment	Clinical registries	United States of America	2001–2009
O’Neill et al. (2016)	Hospital pay-per-performance evaluation	Clinical registries	United Kingdom	2010–2011
Reed et al. (2019)	Health care utilization of patients with chronic conditions	Clinical registries	United States of America	2006–2007
Sofrygin et al. (2017)	Simulation tool of longitudinal and network designs	Simulation models	Not applicable	Not applicable
Sofrygin et al. (2019)	Treatment strategies of diabetes	Electronic health records	United States of America	2001–2009
Zhang et al. (2018)	Treatment strategies of erythropoietin dosing	Electronic health records	United States of America	2006–2010

## Discussion

In the present *Hints and Kinks*, we introduced components for a principled framework for causal inference in HSR. Because ‘causal inference’ is conceptually different from ‘description’ or ‘modeling’, HSR needs the integration of a causal inference framework which includes a specific notation, definitions and analysis techniques to extend the traditional tasks of ‘description’ and ‘modeling’. Public health decision-making which solely relies on associational effect measures might lead to inappropriate decisions because questions about optimal decision-making are inherently causal. We plea that students and researchers in the field of HSR are aware of the different available frameworks to successfully address ‘description’, ‘modeling’ and ‘causal inference’, depending on the intended research question.

## References

[CR1] Danaei G, García Rodríguez LA, Cantero OF (2018). Electronic medical records can be used to emulate target trials of sustained treatment strategies. J Clin Epidemiol.

[CR2] Dickerman BA, García-Albéniz X, Logan RW (2019). Avoidable flaws in observational analyses: an application to statins and cancer. Nat Med.

[CR3] García-Albéniz X, Hsu J, Hernán MA (2017). The value of explicitly emulating a target trial when using real world evidence: an application to colorectal cancer screening. Eur J Epidemiol.

[CR4] Gaughan J, Gutacker N, Grašič K (2019). Paying for efficiency: Incentivising same-day discharges in the English NHS. J Health Econ.

[CR5] Hernán MA (2004). A definition of causal effect for epidemiological research. J Epidemiol Community Health.

[CR6] Hernán M (2018). The C-word: the more we discuss it, the less dirty it sounds. Am J Public Health.

[CR7] Hernán M, Robins J (2020). Causal inference: what if.

[CR8] Héroux J, Moodie EEM, Strumpf E (2014). Marginal structural models for skewed outcomes: identifying causal relationships in health care utilization. Stat Med.

[CR9] Kuehne F, Jahn B, Conrads-Frank A (2019). Guidance for a causal comparative effectiveness analysis emulating a target trial based on big real world evidence: when to start statin treatment. J Comp Eff Res.

[CR10] Moser A, Puhan MA, Zwahlen M (2020). The role of causal inference in health services research I: tasks in health services research. Int J Public Health.

[CR11] Murray EJ, Robins JM, Seage GR (2018). Using observational data to calibrate simulation models. Med Decis Mak.

[CR12] Neugebauer R, Fireman B, Roy JA (2012). Dynamic marginal structural modeling to evaluate the comparative effectiveness of more or less aggressive treatment intensification strategies in adults with type 2 diabetes. Pharmacoepidemiol Drug Saf.

[CR13] O’Neill S, Kreif N, Grieve R (2016). Estimating causal effects: considering three alternatives to difference-in-differences estimation. Heal Serv Outcomes Res Methodol.

[CR14] Pearl J, Glymour M, Jewell NP (2016). Causal inference in statistics: a primer.

[CR15] Reed ME, Huang J, Brand RJ (2019). Patients with complex chronic conditions: health care use and clinical events associated with access to a patient portal. PLoS ONE.

[CR16] Sofrygin O, van der Laan MJ, Neugebauer R (2017). Simcausal R Package: conducting transparent and reproducible simulation studies of causal effect estimation with complex longitudinal data. J Stat Softw.

[CR17] Sofrygin O, Zhu Z, Schmittdiel JA (2019). Targeted learning with daily EHR data. Stat Med.

[CR18] van der Laan MJ, Sherri R (2011). Targeted learning—causal inference for observational and experimental data.

[CR19] Zhang Y, Young JG, Thamer M, Hernán MA (2018). Comparing the effectiveness of dynamic treatment strategies using electronic health records: an application of the parametric g-formula to anemia management strategies. Health Serv Res.

